# Independent Associations between Sedentary Time, Moderate-To-Vigorous Physical Activity, Cardiorespiratory Fitness and Cardio-Metabolic Health: A Cross-Sectional Study

**DOI:** 10.1371/journal.pone.0160166

**Published:** 2016-07-27

**Authors:** Sara Knaeps, Johan Lefevre, Anne Wijtzes, Ruben Charlier, Evelien Mertens, Jan G. Bourgois

**Affiliations:** 1 Physical Activity, Sports and Health Research Group, Department of Kinesiology, KU Leuven, Leuven, Belgium; 2 Department of Movement and Sports Sciences, Ghent University, Ghent, Belgium; 3 Department of Human Biometrics and Biomechanics, Vrije Universiteit Brussel, Brussels, Belgium; University of the Balearic Islands, SPAIN

## Abstract

We aimed to study the independent associations of sedentary time (ST), moderate-to-vigorous physical activity (MVPA), and objectively measured cardiorespiratory fitness (CRF) with clustered cardio-metabolic risk and its individual components (waist circumference, fasting glucose, HDL-cholesterol, triglycerides and blood pressure). We also investigated whether any associations between MVPA or ST and clustered cardio-metabolic risk were mediated by CRF. MVPA, ST, CRF and individual cardio-metabolic components were measured in a population-based sample of 341 adults (age 53.8 ± 8.9 years; 61% men) between 2012 and 2014. MVPA and ST were measured with the SenseWear pro 3 Armband and CRF was measured with a maximal exercise test. Multiple linear regression models and the product of coefficients method were used to examine independent associations and mediation effects, respectively. Results showed that low MVPA and low CRF were associated with a higher clustered cardio-metabolic risk (*β* = -0.26 and *β* = -0.43, both p<0.001, respectively). CRF explained 73% of the variance in the association between MVPA and clustered cardio-metabolic risk and attenuated this association to non-significance. After mutual adjustment for MVPA and ST, CRF was the most important risk factor for a higher clustered cardio-metabolic risk (β = -0.39, p<0.001). In conclusion, because of the mediating role of CRF, lifestyle-interventions need to be feasible yet challenging enough to lead to increases in CRF to improve someone’s cardio-metabolic health.

## Background

According to the World Health Organization, noncommunicable diseases were responsible for 38 million (68%) of the world’s deaths in 2012 [1]. A number of cardio-metabolic risk factors are closely related to these noncommunicable diseases, including visceral obesity, hypertension, hyperglycaemia, and atherogenic dislipidemia [2]. Clustering of these cardio-metabolic risk factors in the same person appears to confer a substantial additional risk for cardiovascular diseases, diabetes mellitus type 2, and all-cause mortality over and above the sum of the risk associated with each abnormality [2, 3]. Over the last few decades, the prevalence of these cardio-metabolic risk factors has increased steadily [4]. Moreover, evidence indicates that in most populations today more than one fourth of the adult population has a clustering of three or more cardio-metabolic risk factors [5].

Evidence from cross-sectional studies [6–10], longitudinal studies [11–15] and randomised controlled trials [16, 17] suggests that sedentary time (ST), moderate-to-vigorous physical activity (MVPA), and cardiorespiratory fitness (CRF) are important predictors of various cardio-metabolic risk factors [18]. However, there are still questions regarding the specificity of these associations and the underlying relationships for predicting clustered cardio-metabolic risk. For example, several studies observed an association between ST and clustered cardio-metabolic risk, independent of physical activity [19–21]. Furthermore, only few studies examined the relationship of all three parameters together for predicting clustered cardio-metabolic risk and results of those studies have been equivocal [22–25]. One study reported that people with a high ST had a 65 to 76% higher risk of developing the metabolic syndrome, although low CRF was the strongest risk factor [22]. In line with these results, another study has identified ST as a risk factor for several markers of cardio-metabolic risk, although the relationship between ST and clustered cardio-metabolic risk was remarkably less pronounced when taking CRF into account [24, 25]. In contrast to these studies, van der Velde et al. observed that not ST, but MVPA and CRF were independently associated with clustered cardio-metabolic risk [23]. Of these studies, only two included objective measurements for MVPA, ST and CRF, however CRF was not measured with a maximal exercise test [23, 25].

Based on the temporal associations of ST and MVPA with CRF [26, 27], it is possible that CRF partly mediates associations between ST, MVPA, and clustered cardio-metabolic risk [28]. In a study by Sassen et al. up to 78% of the association between average physical activity and cardiovascular disease was mediated through CRF [28]. Nevertheless, still 22% of the total variance in physical activity had a direct effect on cardiovascular disease [28], which is important because physical activity is a behaviour that can be changed whereas CRF is a physiological measure reflecting a combination of physical activity behaviors, genetic potential, and functional health of various organ systems [29]. Moreover, to the best of our knowledge, no study has investigated the mediating effect of CRF on the relationship between ST and clustered cardio-metabolic risk.

The aim of the current study was to examine the independent associations of objectively measured ST, MVPA and CRF with clustered cardio-metabolic risk and individual cardio-metabolic risk factors. Furthermore, the mediating effect of CRF on the relation between MVPA or ST and clustered cardio-metabolic risk was analysed. We hypothesized that objectively measured high CRF, high MVPA, and low ST were independently associated with a favourable cardio-metabolic risk profile. Moreover, we hypothesized that CRF is a potential mediator for the association of MVPA or ST and clustered cardio-metabolic risk.

## Methods

### Participants and study design

This cross-sectional study is part of a Flemish longitudinal study of which the sampling procedure is previously described in detail by Matton et al. [30]. Participants (n = 652) were male (n = 420) and female (n = 232) volunteers between 29 and 82 years and were measured between 2012 and 2014 in Leuven, Belgium. For 203 participants (31%) valid physical activity data were not available (8% did not wear the SenseWear Pro 3 Armband and 23% did not comply with the strict inclusion criteria). Furthermore, nine participants (1.4%) did not provide a blood sample and another 89 participants (14%) did not perform a maximal cycle ergometer test. Additionally, 22 participants had missing data for covariates such as nutritional intake, education level and smoking status, leaving a final study sample of 341 participants. Written informed consent was obtained from the participants and the study was approved by the Medical Ethics Committee UZ KU Leuven (s54083).

### Physical activity and sedentary time

Objective measurement of physical activity and ST was obtained with a SenseWear Pro 3 Armband (BodyMedia, Inc, Pittsburgh, PA, USA), which is proven valid for measuring daily energy expenditure under free-living conditions [31]. Participants were asked to wear the SenseWear Pro 3 Armband 24 hours a day, except during water-based activities, for seven consecutive days. The compliance criterion was set at 1296 minutes (90% of a total day). The SenseWear Pro 3 uses algorithms developed by the manufacturer (SenseWear Professional software version 6.1) to combine input from different sensors, including a two-axis accelerometer and sensors measuring heat flux, galvanic skin response, skin temperature, and near body ambient temperature, with information on gender, age, body weight, and height to estimate sleep and wake parameters. Estimated sleeping time was then excluded from all analyses. Furthermore, to achieve more reliable estimates of physical activity and ST, only participants reaching the compliance criterion for at least 3 weekdays and both weekend days were included in the study [32]. Time spent in sedentary behaviour (< = 1.5 MET), light physical activity (LPA, 1.5—< = 3 MET) and MVPA (> 3 MET) was derived using the measured MET values.

### Cardiorespiratory fitness

CRF was determined by means of a maximal exercise test on an electrically braked Lode Excalibur cycle ergometer (Lode, Groningen, the Netherlands). Peak oxygen uptake (VO_2peak_) was measured directly with breath-by-breath respiratory gas exchange analysis, using a Cortex MetaLyzer 3B analyzer (Cortex Biophysic GmbH, Leipzig, Germany) and was defined as the highest 20-s value during the exercise test [33]. The exercise test started at a load of 20 W, which was increased with 20 W every minute until volitional exhaustion.

### Clustered cardio-metabolic risk score

A cardio-metabolic risk score (CMRS), largely based on the International Diabetes Foundation criteria for the metabolic syndrome, was calculated to measure clustered cardio-metabolic risk [2, 14]. A continuous score can better capture the progressive risk increase when more risk factors are present and it has more statistical power than a dichotomous score [34]. Metabolic parameters were assessed by trained staff in the morning after an overnight fast. Waist circumference was measured to the nearest 0.1 cm. Systolic and diastolic blood pressure were measured with an electronic blood pressure monitor (Omron, the Netherlands) three times in seated position. The means of the three measurements for systolic and diastolic blood pressure were used in statistical analyses. Values for triglycerides, plasma glucose and HDL-Cholesterol (HDL-C) were obtained by enzymatic methods (Abbott Laboratories, Abbott Park, IL). Due to skewness in the latter three parameters, values were first normalized (log_10_). To create the CMRS, variables were standardised by subtracting the sex-specific sample means from the individual mean and dividing by the sex-specific standard deviation. Subsequently, these standardised values for waist circumference, blood pressure, triglyceride, plasma glucose, and the inverse of HDL-C were summarized and divided by the number of variables included (x = 5), generating the CMRS. A score above zero represents higher individual cardio-metabolic markers and therefore indicates having a less preferable clustered cardio-metabolic health.

### Covariates

Smoking behaviour was assessed using the WHO Monica Smoking Questionnaire [35]. Participants were classified as current, former or never smokers. Four levels of education were ranked from lowest (no degree) to highest (university degree) and were used as an indicator of socio-economic status [36].

Sugar intake, saturated fat intake and alcohol consumption were assessed using a three-day diet record, during two weekdays and one weekend day. When possible all foods and drinks were weighed and recorded. Otherwise they were estimated using standard household measures such as a plate, spoon or glass. The diet records were analysed using Becel Nutrition software (Unilever Co., Rotterdam, the Netherlands).

### Statistical analysis

Descriptive statistics (median and interquartile range) were calculated for all participants. Pearson correlation coefficients were calculated between all cardio-metabolic risk factors and the three main exposures; ST, MVPA, and CRF. Additionally, Pearson correlations were calculated between the three exposure variables (S1 Table).

Furthermore, multiple linear regression was used to examine the association of ST, MVPA and CRF with cardio-metabolic risk. Residuals were tested for homoscedasticity, linearity and independence. Additionally, the variance inflation factor never exceeded three, indicating that multi-collinearity was not a concern [37].

Both exposure and outcome variables were standardized in order to enable direct comparison of the effect estimates across outcome and exposure variables, and regression coefficients (95% CI) are presented. All models were initially adjusted for age, sex, waking time, smoking, alcohol consumption, sugar and saturated fat intake, and education level (Model 1). Subsequently, Model 1 was further separately adjusted for ST and MVPA (Model 1 + ST, Model 1 + MVPA). Next, a fully adjusted model with all three exposures was composed for ST, MVPA and CRF (Model 2). Regression coefficients yielded from analyses using unstandardized exposures and outcomes are presented in S2 Table.

Finally, mediation analyses were performed to assess whether CRF is a mediator in the associations of ST and MVPA with CMRS. Mediation analyses were performed only when ST or MVPA were independently associated with CMRS (i.e. Model 1: adjusted for covariates and each other). Mediation was established by the use of regression analyses of unstandardized exposures and outcomes including the following steps: 1) regressing the outcome (CMRS) on the exposure (ST/MVPA) (path c), 2) regressing the mediator (CRF) on the exposure (path a), 3) regressing the outcome on the mediator, adjusted for the exposure (path b). All regression analyses were adjusted for covariates and the other exposure (i.e. for ST in case of MVPA and for MVPA in case of ST). Size of the mediated effect was estimated by the product of coefficients method (a*b) by MacKinnon et al [38]. Proportion mediated was calculated by dividing the product of coefficients by the overall effect (a*b/c). The mediated effect was tested by the Sobel test [39].

Statistical analyses were performed using the SAS, version 9.4 (SAS institute, Cary, NC, USA) statistical program. Statistical significance was set at p < .05.

## Results

Table 1 presents descriptive statistics for MVPA, ST, CRF, and cardio-metabolic markers. The mean age of the participants was 53.8 ± 8.9 years. In total 207 men and 134 women (39%) were included, of whom most were former smokers (34%) or never smokers (58%). In general the study population was highly educated with more than 95% having finished secondary schooling. The mean alcohol consumption of the participants was 13.4±10.2 mg/day, which is an average of one standard drink per day. Mean sugar intake was 122±47.5 mg/day and mean saturated fat intake was 32.8±12.7 mg/day. When compared to non-participants (n = 311), participants had similar MVPA, ST, CRF and CMRS. However, participants were on average 5 years younger.

**Table 1:** Descriptive statistics for age, physical activity, physical fitness and cardio-metabolic markers.

	Total (*n* = 341)	
Variables	*M*	*Q1-Q3*
Age (years)	53.9	49.2–57.7
BMI (kg/m^2^)	24.3	22.8–26.4
Sedentary time (hours/day)	10.8	9.6–12.3
LPA (hours/day)	3.8	3.2–4.7
MVPA (hours/day)	2.1	1.4–3.4
VO_2_peak (ml.min^-1^.kg^-1^)	32	28–39
Cardio-metabolic markers		
Waist Circumference (cm)	81.1	77.9–91.2
Fasting Glucose (mmol/L)	5.06	4.78–5.39
HDL-cholesterol (mmol/L)	1.47	1.27–1.76
Triglycerides	0.99	0.76–1.37
Diastolic Blood Pressure (mmHg)	84.5	79.3–91.0
Systolic Blood Pressure (mmHg)	131.8	121.7–142.0

Note. *M* = median, Q1 = lower quartile, Q3 = upper quartile

LPA = Light physical activity, MVPA = Moderate-to-vigorous physical activity.

ST had a high negative correlation with MVPA (*r* = -.71, *p* = < .001), however not with CRF (*r* = -.09, *p* = .11). Furthermore MVPA had a moderate positive correlation with CRF (*r* = .43, *p* = < .001). Pearson correlation coefficients for the association between ST, MVPA, CRF and cardio-metabolic markers are shown in S1 Table. In general, correlations were low to moderate, ranging from -0.42 to 0.27. As hypothesized, clustered cardio-metabolic risk was positively associated with ST and negatively with MVPA and CRF.

Results from multiple linear regression analyses using standardized and unstandardized exposure and outcome variables can be found in Table 2 and S2 Table, respectively. ST was positively associated with waist circumference and negatively with HDL-C (Model 1). After adjustment for MVPA (Model 1 + MVPA) and both MVPA and CRF together (Model 2), these associations remained significant (Table 2).

**Table 2:** Standardized regression coefficients of sedentary behavior, moderate-to-vigorous physical activity and cardiorespiratory fitness for clustered cardio-metabolic health and waist circumference.

		ST			MVPA			CRF		
	Model			95% CI			95% CI			95% CI
CMRS	Model 1	0.12		-0.00, 0.23	-0.26	***	-0.37, -0.15	-0.43	***	-0.53, -0.32
	Model 1 + ST				-0.35	***	-0.50, -0.21	-0.43	***	-0.54, -0.33
	Model 1 + MVPA	-0.14		-0.30, 0.01				-0.38	***	-0.49, -0.27
	Model 2	0.05		-0.11, 0.21	-0.10		-0.26, 0.06	-0.39	***	-0.52, -0.27
Waist Circumference	Model 1	0.24	***	0.14, 0.35	-0.22	***	-0.32, -0.12	-0.04		-0.14, 0.07
	Model 1 + ST				-0.12		-0.26, 0.01	-0.04		-0.14, 0.06
	Model 1 + MVPA	0.15	*	0.01, 0.30				0.05		-0.06, 0.16
	Model 2	0.15		0.04, 0.30	-0.13		-0.29, 0.03	-0.01		-0.11, 0.13
Fasting Glucose	Model 1	0.03		-0.09, 0.14	-0.01		-0.13, 0.10	-0.03		-0.15, 0.08
	Model 1 + ST				0.00		-0.15, 0.16	-0.03		-0.15, 0.08
	Model 1 + MVPA	0.03		-0.14, 0.19			,	-0.03		-0.15, 0.09
	Model 2	0.05		-0.13, 0.23	0.03		-0.14, 0.21	-0.04		-0.18, 0.09
HDL-cholesterol	Model 1	-0.28	***	-0.39, -0.16	0.09		-0.03, 0.20	-0.15	*	-0.26, -0.03
	Model 1 + ST			,	-0.18		-0.33, 0.03	-0.14	*	-0.26, -0.03
	Model 1 + MVPA	-0.41	***	-0.57, -0.25			,	-0.20	**	-0.33, -0.08
	Model 2	-0.36	***	-0.53, -0.18	-0.11		-0.29, 0.06	-0.10		-0.23, 0.03
Triglycerides	Model 1	0.09		-0.03, 0.21	-0.14	*	-0.25, -0.03	-0.20	**	-0.32, -0.08
	Model 1 + ST			,	-0.16	*	-0.32, -0.00	-0.20	***	-0.32, -0.08
	Model 1 + MVPA	-0.03		-0.20, 0.14			,	-0.17	**	-0.29, -0.04
	Model 2	0.06		-0.12, 0.24	-0.04		-0.22, 0.14	-0.18	*	-0.32, -0.05
Diastolic Blood Pressure	Model 1	0.06		-0.06, 0.17	-0.01		-0.12, 0.10	-0.11	*	-0.23, -0.02
	Model 1 + ST			,	0.05		-0.10, 0.21	-0.11		-0.23, 0.00
	Model 1 + MVPA	0.10		-0.07, 0.03			,	-0.12	*	-0.25, -0.00
	Model 2	0.18	**	0.01, 0.36	0.17		0.00, 0.34	-0.18	**	-0.31, -0.04
Systolic Blood Pressure	Model 1	0.06		-0.05, 0.17	0.07		-0.03, 0.18	0.05		-0.05, 0.16
	Model 1 + ST			,	0.22	**	0.08, 0.36	0.05		-0.05, 0.16
	Model 1 + MVPA	0.22	**	0.07, 0.37				0.03		-0.08, 0.15
	Model 2	0.24	**	0.08, 0.40	0.24	**	0.08, 0.41	-0.04		-0.16, 0.09

Data are standardized regression coefficients

ST = Sedentary Time; MVPA = moderate-to-vigorous physical activity; CRF = Cardiorespiratory fitness; CMRS = Cardio-metabolic risk score

Model 1: adjusted for age, sex, original study population, smoking, education level, alcohol intake, sugar and saturated fat intake and waking time

Model 2: adjusted for all covariates in model 1 and adjusted for ST, MVPA and CRF as applicable

*p<0.05;

**p<0.01;

***p<0.001

MVPA was negatively associated with CMRS, triglycerides and waist circumference (Model 1). After adjustment for ST (Model 1 + ST), this association was of similar strength for CMRS. Furthermore, a new association with systolic blood pressure became apparent. When adjusting for both ST and CRF together (Model 2), all associations attenuated to non-significance, with the exception of the association with systolic blood pressure.

CRF was negatively associated with CMRS, triglycerides and diastolic blood pressure, and positively associated with HDL-C (Model 1). Associations with CMRS, HDL-C and triglycerides remained significant after adjustment for ST or MVPA separately. Furthermore, all initial associations from Model 1 remained significant after adjustment for both ST and MVP (Model 2), with the exception of the association with HDL-C.

**Fig 1:**
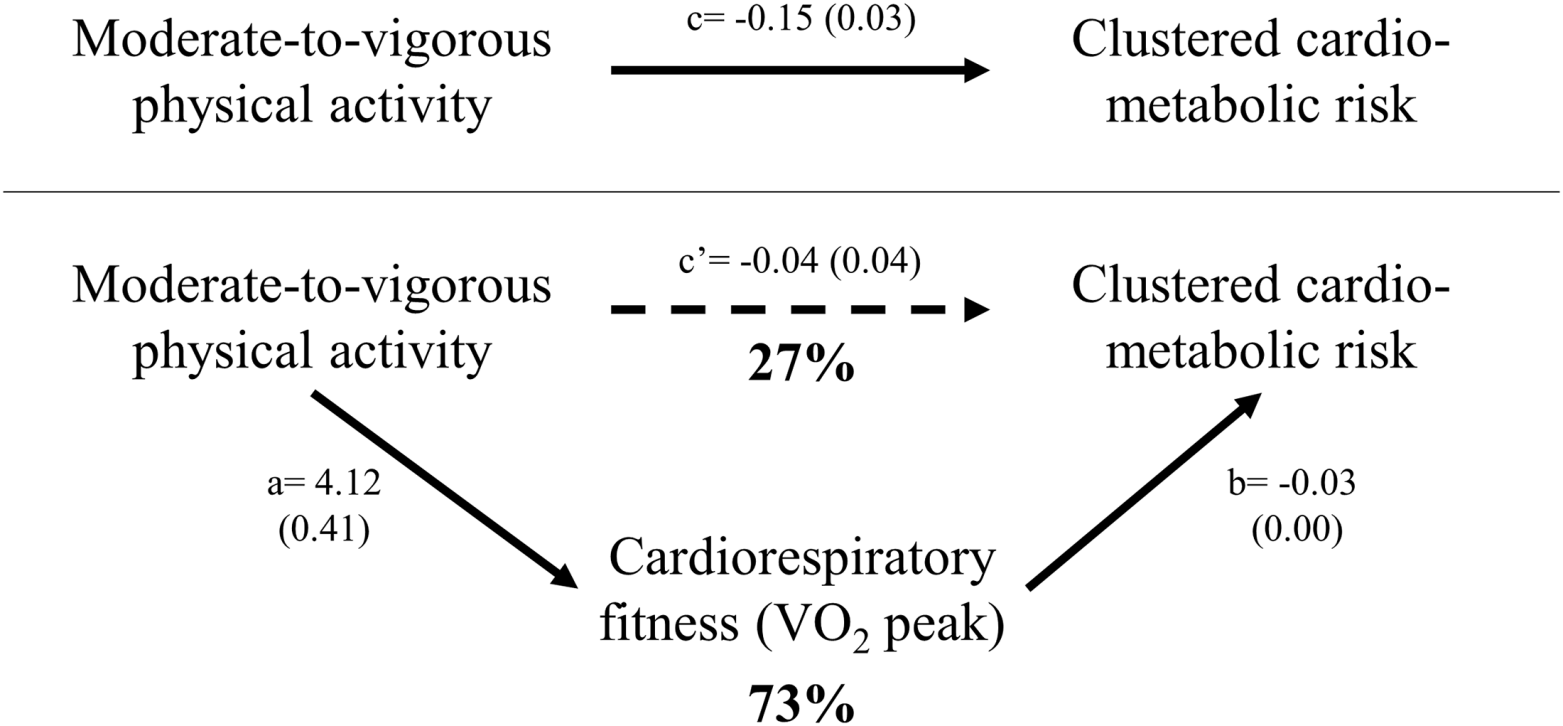
Mediation pathway of cardiorespiratory fitness. Pathway (regression coefficient of unstandardized exposures and outcomes (standard error)) of the association between moderate-to-vigorous physical activity (MVPA, hours/day) and clustered cardio-metabolic risk (CMRS) through cardiorespiratory fitness (CRF, ml.min^-1^.kg^-1^) corrected for all covariates: sedentary time, age, sex, waking time, smoking, alcohol consumption, suger intake, saturated fat intake and education level. CRF explains 73% of the total variance in CMRS.

Finally, we investigated the mediated effect of CRF on the association between MVPA and CMRS. There was significant mediation by CRF in the association between MVPA and CMRS, (a*b (95% CI): -0.11 (-0.11; -0.10), *p* < 0.001). The mediated effect was calculated [4.12 * -0.03 / -0.15] * 100%; resulting in an effect of 73%, meaning that CRF explained 73% of the total variance in the association between MVPA and CMRS (Fig 1). Because there was no association between ST and CMRS (i.e. no total effect), no further mediation analysis was performed for this association.

## Discussion

The main findings of this cross-sectional study suggest that low MVPA is a significant exposure for clustered cardio-metabolic risk and that low ST is a significant exposure for a healthier waist circumference and HDL-C. Low CRF appeared to be the most important exposure for a higher clustered cardio-metabolic risk and is an important mediator in the association between MVPA and CMRS. Contrary to our research hypothesis, only CRF was associated with clustered cardio-metabolic risk independent from the other two potential exposures, MVPA and ST. However, when only examining waist circumference, ST appeared to be a more important predictor than MVPA or CRF.

Although a growing number of studies have examined the associations of ST and/or physical activity and/or CRF [7, 8, 12, 14, 18], with clustered cardio-metabolic risk, few have taken into account all three exposures simultaneously. Moreover, even fewer studies have used objective measurement for all three exposures or analysed the mediating influence of CRF [22–25, 28]. Regression coefficients from analyses using unstandardized exposure and outcome variables illustrate the clinical significance of the associations. Even in this healthy population, where 268 (79%) had ≤ 1 at risk measurements for cardio-metabolic risk factors (according to the guidelines of the International Diabetes Foundation [2]) and only 16 participants (5%) had at risk measurements for ≥ 3 cardio-metabolic risk factors, significant improvements can be achieved given that after correction for MVPA one hour less ST was associated with a 0.83 cm lower waist circumference.

Similar previous studies including all three exposures underline the importance of high CRF and support the conclusion of an independent association between CRF and clustered cardio-metabolic risk [22–24]. However, contrary to the present study, all studies found an inverse association between sedentary behaviour and cardio-metabolic risk, though in only two studies this association remained significant after adjusting for physical activity and CRF [22, 25]. Moreover, in the present study, the lack of an association between MVPA and clustered cardio-metabolic risk independent of ST and CRF appears to be in contradiction with the results of van der Velde et al. [23].

Because studies incorporating all three exposures are rather scarce, results are also compared to studies including only two exposures. For example, inconsistent findings have been reported for the association between ST and clustered cardio metabolic risk when adjusting for MVPA or CRF [9, 19, 21]. We found no evidence for an association between ST and clustered cardio-metabolic risk. Similar results have been shown by van der Velde et al. [23] and Scheers et al. [6] who found that the inverse relationship between ST and the metabolic syndrome could not be maintained after adjusting for MVPA and/or CRF. However, some studies did observe an association between ST and clustered cardio-metabolic risk independent of MVPA [14, 19, 21]. Age might be an important moderator of the independent relationship between ST and cardio-metabolic risk. Studies with participants with an average age over 60 reported ST as an important independent predictor [19, 21], while studies in younger populations did not find an independent association between ST and clustered cardio-metabolic risk [6, 23]. Moreover, Maher et al. suggest that when adjusting for total physical activity instead of only MVPA, there is virtually no association between ST and cardio-metabolic risk factors [40].

No association between MVPA and clustered cardio-metabolic risk was found independent of both ST and CRF. This supports the hypothesis that CRF mediates the association between MVPA and clustered cardio-metabolic risk because we observed a significant association independent of ST only. In comparison, some longitudinal studies argue that a higher physical activity may lower clustered cardio-metabolic risk even in the absence of an improvement of CRF [12, 17, 41] or high ST [6]. Furthermore, a training study investigating the effect of MVPA on blood lipids and CRF observed an improved blood lipid profile in spite of a decrease in CRF [17]. However, in a study with a population of recently diagnosed type 2 diabetes patients, the observed association between MVPA and clustered cardio-metabolic risk reduced to a non-significant level after accounting for ST [21].

Our findings support the hypothesis that CRF is a significant exposure for cardio-metabolic risk and an important mediator in the association between MVPA and clustered cardio-metabolic risk. This result is in line with previous cross-sectional research [7, 28, 42, 43]. A study with more than 1500 participants concluded that both physical activity, but mainly, CRF were independently associated with lower clustered cardio-metabolic risk [44]. Moreover, Franks et al. observed a strong inverse relationship between CRF and clustered cardio-metabolic risk, and MVPA and clustered cardio-metabolic risk [8]. However, CRF modified the association between physical activity and clustered cardio-metabolic health to such an extent that in fit people the relationship did not exist [8]. Although CRF is often observed as the most important independent exposure, it is important to note that CRF can be genetically predisposed up to 47% and as such is not completely modifiable [45]. Moreover, the effect estimate of MVPA, comparable in size with the study by Sassen et al. [46], was not completely attenuated after adjustment for CRF (30% unexplained), indicating that there may be other pathways relating MVPA with cluster cardio-metabolic risk. In addition, multiple studies have shown the importance of physical activity and/or sedentary behavior for improving CRF [26, 47].

Strengths of this study include the objective measurement of ST and MVPA. Objective measurement over a substantial time period provides a more accurate and complete insight into the participants’ physical activity and ST [32]. Moreover, we utilised strict inclusion criteria (90% wear time per day and 3 week days plus both weekend days) to gather the most comprehensive interpretation of ST and MVPA. It should be noted that the amount of MVPA in the current study is higher than reported in previous studies, however similar to a comparable Flemish population [6], this might be due to the specific algorithms that SenseWear Pro 3 uses. It is possible that the recording of MVPA and ST over a 24hr period and in single minutes instead of in bouts (e.g. bouts of 10 min) may have led to a more comprehensive measurement of MVPA. Alternatively, our study sample may have included healthier, more active, individuals compared to the average population Finally, CRF was measured by a maximal cycle ergometer exercise test, generally considered the gold standard [48], leaving less room for measurement error.

However, this study is not without limitations. First, the cross-sectional nature of the data does not allow inferences of causality. Second, the SenseWear Pro 3 Armband probably underestimates total energy expenditure, particularly at higher intensities [31]. However, given that the current study uses minutes per day above 3 MET to define minutes spent in MVPA, and not the energy expenditure during MVPA, it is unlikely that this has affected our results. Additionally, the SenseWear Pro 3 Armband cannot measure posture and therefore standing may have been included in ST. Third, medication use was not assessed and therefore not controlled for in analyses. Although the study population is rather healthy, this may have resulted in residual confounding. Fourth, a substantial number of participants were excluded from the analyses due to the absence of CRF data or SenseWear Pro 3 data. A drop-out analysis confirmed our assumption that, for SenseWear Pro 3 data, not complying with the inclusion criteria was a random event and therefore did not influence the results. However, the most important reason for not completing the maximal cycle ergometer test was exclusion by a physician. Most common reasons for exclusion were lower back pain, a higher risk of myocardial infarction, arterial hypertension, abnormalities on an electrocardiogram, or clinical judgment. Because of these exclusion criteria, the least fit participants were probably excluded, resulting in a more fit and therefore homogenous sample.

Finally, comparison of results from different studies is often difficult due to differences in measurements (e.g. objective measurements versus subjective measurements), variable definitions, or study populations. For example, physical activity is often defined differently (e.g. habitual physical activity [8], leisure-time physical activity [22], overall energy expenditure [11], physical activity energy expenditure [12, 41], total physical activity [40] or MVPA [6, 23]) and as a consequence, not all studies deal with the same intensities and volumes of physical activity. In a similar vein, although most studies have investigated sedentary behaviour or ST, only few look at the whole range of sedentary behaviours or ST over a 24h period of time [23, 24]. Moreover, ST is frequently based on self-report, only including television viewing [15, 49, 50], passive transportation, or both [22, 24]. With regard to the study population, it is plausible that not only age, race or gender but also health status of the population influences associations with cardio-metabolic risk. For example, the present study presents a relatively healthy and highly educated group of adults.

## Conclusions

In summary, CRF appears to be the most important exposure (in comparison to ST and MVPA) for clustered cardio-metabolic risk. This study also highlights some important associations between ST, MVPA and CRF, and certain cardio-metabolic risk factors. Furthermore, our findings suggest that the risk related to low MVPA is substantially mediated by CRF. Therefore, when designing interventions for reducing cardio-metabolic risk, apart from an increase in MVPA and decrease in ST, an increase in CRF will have to be the additional goal. Consequently, exercise programs need to be feasible yet challenging enough to lead to increases in CRF.

## Supporting Information

S1 Table Pearson correlation coefficients for the association between physical activity, sedentary time, physical fitness and cardio-metabolic markers. (DOCX)

S2 Table Unstandardized regression coefficients of sedentary behavior, moderate-to-vigorous physical activity and cardiorespiratory fitness for cardio-metabolic markers. (DOCX)
