# Supplementary material for: Independent Associations between Sedentary Time, Moderate-To-Vigorous Physical Activity, Cardiorespiratory Fitness and Cardio-Metabolic Health: A Cross-Sectional Study
Source: PLoS One. 2016 Jul 27;11(7):e0160166. doi: 10.1371/journal.pone.0160166 (PMC4963092; doi:10.1371/journal.pone.0160166)
Supplement: S1 Table — (DOCX) [file pone.0160166.s001.docx]

| **Supplement 1. Pearson correlation coefficients for the association between physical activity, sedentary time, physical fitness and cardio-metabolic markers** | | | |
| --- | --- | --- | --- |
|  | ST | MVPA | CRF |
| CMRS | 0.13* | -0.30*** | -0.42*** |
| Waist Circumference | 0.29*** | -0.26*** | -0.07 |
| Fasting Glucose | 0.03 | -0.08 | -0.12* |
| HDL-cholesterol | -0.27*** | 0.06 | 0.16** |
| Triglycerides | 0.06 | -0.14** | -0.15** |
| Diastolic Blood Pressure | 0.13* | 0.00 | -0.05 |
| Systolic Blood Pressure | 0.12* | -0.04 | -0.10 |
| * p < 0.05, ** p < 0.01, *** p < 0.001 | | | |
| ST = Sedentary Time, MVPA = Moderate-to-vigorous physical activity, CRF = Cardiorespiratory fitness, CMRS = Cardio-metabolic risk score | | | |
