# Supplementary material for: Independent Associations between Sedentary Time, Moderate-To-Vigorous Physical Activity, Cardiorespiratory Fitness and Cardio-Metabolic Health: A Cross-Sectional Study
Source: PLoS One. 2016 Jul 27;11(7):e0160166. doi: 10.1371/journal.pone.0160166 (PMC4963092; doi:10.1371/journal.pone.0160166)
Supplement: S2 Table — (DOCX) [file pone.0160166.s002.docx]

| **Supplement 2. Unstandardized regression coefficients of sedentary behavior, moderate-to-vigorous physical activity and cardiorespiratory fitness for cardio-metabolic markers** | | | | | | | | | | | | | | | | | | |
| --- | --- | --- | --- | --- | --- | --- | --- | --- | --- | --- | --- | --- | --- | --- | --- | --- | --- | --- |
|  |  | ST (hours/day) | | | | |  | MVPA (hours/day) | | | | |  | CRF (ml.min-1.kg-1) | | | | |
|  | Model |  |  | 95% CI | | |  |  |  | 95% CI | | |  |  |  | 95% CI | | |
| CMRS | Model 1 | 0.04 |  | -0.00 | , | 0.08 |  | -0.11 | *** | -0.15 | , | -0.06 |  | -0.03 | *** | -0.03 | , | -0.02 |
|  | Model 1 + ST |  |  |  | , |  |  | -0.15 | *** | -0.21 | , | 0.00 |  | -0.03 | *** | -0.04 | , | -0.02 |
|  | Model 1 + MVPA | 0.00 |  | -0.10 | , | 0.00 |  |  |  |  | , |  |  | -0.02 | *** | -0.03 | , | -0.02 |
|  | Model 2 | 0.02 |  | -0.04 | , | 0.07 |  | -0.04 |  | -0.11 | , | 0.03 |  | -0.03 | *** | -0.03 | , | -0.02 |
| Waist Circumference (cm) | Model 1 | 1.33 | *** | 0.75 | , | 1.86 |  | -1.50 | *** | -2.18 | , | -0.81 |  | -0.04 |  | -0.16 | , | 0.08 |
|  | Model 1 + ST |  |  |  | , |  |  | -0.85 |  | -1.61 | , | 0.09 |  | -0.04 |  | -0.16 | , | 0.07 |
|  | Model 1 + MVPA | 0.83 | * | 0.03 | , | 1.62 |  |  |  |  | , |  |  | 0.06 |  | -0.06 | , | 0.18 |
|  | Model 2 | 0.80 |  | -0.05 | , | 1.66 |  | -0.89 |  | -1.98 | , | 0.19 |  | 0.01 |  | -0.12 | , | 0.14 |
| Fasting Glucose | Model 1 | 0.01 |  | -0.03 | , | 0.04 |  | -0.01 |  | -0.05 | , | 0.04 |  | -0.00 |  | -0.01 | , | 0.00 |
|  | Model 1 + ST |  |  |  | , |  |  | 0.00 |  | -0.06 | , | 0.06 |  | -0.00 |  | -0.01 | , | 0.01 |
|  | Model 1 + MVPA | -0.01 |  | -0.04 | , | 0.06 |  |  |  |  | , |  |  | -0.00 |  | 0.00 | , | 0.01 |
|  | Model 2 | 0.02 |  | -0.04 | , | 0.07 |  | 0.01 |  | -0.05 | , | 0.08 |  | 0.00 |  | -0.01 | , | 0.01 |
| HDL-cholesterol | Model 1 | -0.06 | *** | -0.08 | , | -0.03 |  | 0.02 |  | -0.02 | , | 0.01 |  | -0.01 | * | 0.01 | , | -0.00 |
|  | Model 1 + ST |  |  |  | , |  |  | -0.05 |  | -0.09 | , | -0.01 |  | -0.01 | * | -0.01 | , | -0.00 |
|  | Model 1 + MVPA | -0.09 | *** | -0.12 | , | -0.05 |  |  |  |  | , |  |  | -0.01 | ** | -0.01 | , | -0.00 |
|  | Model 2 | -0.08 | *** | -0.11 | , | -0.04 |  | -0.03 |  | -0.08 | , | 0.02 |  | 0.00 |  | -0.01 | , | 0.00 |
| Triglycerides | Model 1 | 0.03 |  | -0.01 | , | 0.07 |  | -0.06 | * | -0.11 | , | -0.01 |  | -0.01 | *** | -0.02 | , | -0.01 |
|  | Model 1 + ST |  |  |  | , |  |  | -0.07 | * | -0.14 | , | -0.00 |  | -0.01 | *** | -0.02 | , | -0.01 |
|  | Model 1 + MVPA | -0.01 |  | -0.07 | , | 0.05 |  |  |  |  | , |  |  | -0.01 | ** | -0.02 | , | -0.00 |
|  | Model 2 | 0.02 |  | -0.04 | , | 0.09 |  | -0.02 |  | -0.10 | , | 0.06 |  | -0.01 | ** | -0.02 | , | 0.00 |
| Diastolic Blood Pressure (mmHg) | Model 1 | 0.28 |  | -0.30 | , | 0.86 |  | -0.05 |  | -0.72 | , | 0.63 |  | -0.11 | * | -0.21 | , | 0.01 |
|  | Model 1 + ST |  |  |  | , |  |  | 0.34 |  | -0.61 | , | 1.29 |  | -0.11 |  | -0.23 | , | 0.00 |
|  | Model 1 + MVPA | 0.48 |  | -0.33 | , | 1.28 |  |  |  |  | , |  |  | -0.12 | * | -0.24 | , | -0.00 |
|  | Model 2 | 0.91 | * | 0.05 | , | 1.77 |  | 1.06 |  | -0.03 | , | 1.77 |  | -0.17 | ** | -0.30 | , | -0.04 |
| Systolic Blood Pressure (mmHg) | Model 1 | 0.49 |  | -0.40 | , | 1.39 |  | 0.80 |  | -0.26 | , | 1.86 |  | 0.09 |  | -0.09 | , | 0.27 |
|  | Model 1 + ST |  |  |  | , |  |  | 1.97 | ** | 0.82 | , | 3.73 |  | 0.09 |  | -0.09 | , | 0.26 |
|  | Model 1 + MVPA | 1.82 | ** | 0.60 | , | 3.05 |  |  |  |  |  |  |  | 0.05 |  | 0.14 | , | 0.24 |
|  | Model 2 | 1.98 | ** | 0.65 | , | 3.30 |  | 2.52 | ** | 0.85 | , | 4.20 |  | -0.06 |  | -0.26 | , | 0.14 |
| Data are unstandardized regression coefficients | | | | | | | | | | | | | | | | | | |
| ST = Sedentary Time; MVPA = moderate-to-vigorous physical activity; CRF=Cardiorespiratory fitness; CMRS = Cardio-metabolic risk score | | | | | | | | | | | | | | | | | | |
| Model 1: adjusted for age, sex, original study population, smoking, education level, alcohol intake, sugar and saturated fat intake, waking time | | | | | | | | | | | | | | | | | | |
| Model 2: adjusted for all covariates in model 1 and adjusted for ST, MVPA and CRF as applicable | | | | | | | | | | | | | | | | | | |
| *p<0.05; **p<0.01; ***p<0.001 | | | | | | | | | | | | | | | | | | |
